# Iron stores in steady‐state sickle cell disease children accessing care at a sickle cell disease clinic in Kumasi, Ghana: A cross‐sectional study

**DOI:** 10.1002/hsr2.934

**Published:** 2022-11-24

**Authors:** Ernest Amanor, Alexander Kwarteng, Amma Larbi, Fatima Amponsah Fordjour, Kelvin Kwaku Koranteng, David Sebbie Sackey, Emmanuel Bannor, Francis Adjei Osei, Aliyu Mohammed, Ezekiel Bonwin Ackah, Samuel Frimpong Odoom, Samuel Blay Nguah, Vivian Paintsil, Alex Osei‐Akoto

**Affiliations:** ^1^ Department of Biochemistry and Biotechnology, College of Science Kwame Nkrumah University of Science and Technology Kumasi Ghana; ^2^ Tropical Infections and Non‐communicable Disease Research Group Kumasi Center for Collaborative Research in Tropical Medicine Kumasi Ghana; ^3^ Department of Hematology, Laboratory Services Directorate Komfo Anokye Teaching Hospital Kumasi Ghana; ^4^ Department of International Health, School of Public Health Kwame Nkrumah University of Science and Technology Kumasi Ghana; ^5^ Department of Biostatistics and Epidemiology, School of Public Health Kwame Nkrumah University of Science and Technology Kumasi Ghana; ^6^ Department of Medical Diagnostics, School of Public Health Kwame Nkrumah University of Science and Technology Kumasi Ghana; ^7^ Child Health Directorate Komfo Anokye Teaching Hospital Kumasi Ghana; ^8^ Department of Child Health, School of Medicine and Dentistry Kwame Nkrumah University of Science and Technology Kumasi Ghana

**Keywords:** complete blood count, hemotransfusion, iron stores, red cell indices, serum ferritin, sickle cell disease

## Abstract

**Background and Aims:**

Children with sickle cell disease (SCD) have an increased risk of multiple hemotransfusions and this can predispose them to elevated iron stores. The objectives of the study were to determine the extent of elevated iron stores and the associated risk factors in a population of steady‐state SCD children in Ghana.

**Methods:**

This cross‐sectional study was conducted at the pediatric sickle cell clinic at the Komfo Anokye Teaching Hospital. Complete blood count and serum ferritin assay were performed for (*n* = 178) steady‐state SCD children. Descriptive and multivariate logistic regression analysis were performed. Elevated iron stores were defined as serum ferritin levels >300 ng/ml. Statistical significance was considered at *p* < 0.05.

**Results:**

The mean (standard deviation) age of the participants was 9.61 (±4.34) years, and 51% of them were males. About 17% of SCD children had elevated iron stores and receiving at least three hemotransfusions during the last 12 months was strongly associated with elevated iron stores (*p* < 0.001). History of chronic hemotransfusion increased the odds of having elevated iron store (adjusted odds ratio [aOR] = 11.41; 95% confidence interval [CI] = 3.11–30.85; *p* < 0.001) but SCD patients on hydroxyurea treatment had reduced‐odds of having elevated iron stores (aOR = 0.18; 95% CI = 0.06–0.602; *p* = 0.006). Moreover, red blood cell (Coef. = −0.84; 95% CI = −0.37, −1.32; *p* = 0.001), hemoglobin (Coef. = −0.83; 95% CI = −0.05, −1.61; *p* = 0.04), hematocrit (Coef. = −0.85; 95% CI = −0.08, −1.63; *p* = 0.03), mean cell volume (Coef. = 0.02; 95% CI = 0.01, 0.03; *p* = 0.001) and mean cell hemoglobin (Coef. = 0.04; 95% CI = 0.01, 0.07; *p* = 0.002) could significantly predict serum ferritin levels.

**Conclusion:**

The magnitude of elevated iron stores was high among children with SCD in steady‐state. Red cell indices could provide invaluable information regarding the risk of elevated iron stores. SCD children who have a history of chronic hemotransfusion or had received at least three hemotransfusions in a year should be monitored for elevated iron stores.

## 1 INTRODUCTION

Sickle cell disease (SCD) is a chronic genetic disorder that results from the inheritance of two abnormal copies of hemoglobin from carrier parents. SCD presents in several phenotypes such as the severe homozygous form (HbSS) and the less symptomatic heterozygous variants (HbSC, HbSD, HbSO‐Arab, and HbS/beta‐thalassemia)^1^, ^2^ These abnormal hemoglobins are due to a single amino acid substitution in the β‐globin chain of normal haemoglobin (HbA) i.e., glutamic acid is replaced with valine or lysine in the 6th position of the β‐globin chain, thereby producing abnormal HbS or HbC, respectively^3^, ^4^ At low oxygen tension, HbS polymerizes and converts the flexible biconcave red blood cells (RBCs) into irreversible rigid, sickle‐shaped RBCs or RBCs crystalized in the case of HbC that obstructs blood flow in the microcirculation.^2^, ^3^, ^4^ This causes clinical symptoms such as vaso‐occlusive pain episodes (VOPE), severe pallor, dactylitis, acute chest syndrome (ACS), stroke, and organ injuries which often warrant hospital admissions for disease management^2^, ^4^, ^5^

Every year, over 300,000 babies are born with SCD worldwide; and approximately 5% of the global population are healthy carriers of the gene for SCD^1^, ^6^ In Africa, over 200,000–300,000 children born every year have SCD, with 75%–80% of these children found in sub‐Saharan Africa^1^, ^6^, ^7^, ^8^ Furthermore, it is reported that only about 20% of newborns with SCD survive to their second birthday in Africa^3^, ^9^ Until recently that it emerged, SCD can be cured, the disease has been managed with a medication regimen of vitamins, folic acid, penicillin V, and hydroxyurea (mostly in HbSS patients) every day^8^, ^10^ However, hemotransfusion therapy has also been accepted as the standard therapy for the management of severe cases such as anemia, ACS and stroke.^11^, ^12^, ^13^ While this therapy effectively reduces the risk of SCD complications, elevated iron stores or iron overload is a dreaded and inevitable clinical consequence of multiple transfusions^3^, ^14^ For every unit of whole blood transfused, the body receives about 200–250 mg of iron^15^, ^16^ Besides, iron is disproportionately released into the body from chronic hemolysis and increased absorption of iron from the gastrointestinal tract^17^, ^18^ Since the body has no excretory mechanism for the excess iron^17^, ^18^ it accumulates which causes elevated iron stores or iron overload. The accumulation of excess iron in tissues or organs produces reactive oxygen species in the cytoplasm through the Fenton and Haber‐Weiss reaction, which consequently lead to mitochondrial damage, disruption of the electron transport chain, peroxidation of lipids and cell membrane damage^19^, ^20^ This eventually causes apoptosis of the target organs with time, leading to complications such as endocrine, liver and heart failure, which is the commonest cause of death due to iron overload.^19^, ^21^

Generally, it is reported that patients who are hemotransfused with at least 10 units of blood are at significant risk of elevated iron stores or iron overload^16^, ^22^ Nonetheless, findings from several studies show that receiving at least three hemo transfusions per year by a sickle cell patient is significantly associated with elevated iron stores.^23^, ^24^, ^25^ Elevated iron stores remain a burden in several parts of Africa, particularly holoendemic malaria settings. Cross‐sectional studies conducted among 70 Congolese^25^ and 85 Nigerian^23^ children with SCD showed that nearly 21% had iron overload. In a retrospective study to analyze the clinical and/or autopsy findings of 141 adult SCD patients at postmortem in Howard University, Washington, DC, about one‐third of the patients had iron overload and 7% of deaths were associated with iron overload.^26^ Elsewhere in Atlanta, Georgia, a study retrospectively reported that 22 out of 387 young adults with SCD died and 10 (45%) of the deaths caused by chronic organ failure were all due to chronic iron overload (end‐stage liver disease in 8 patients and congestive heart failure in 2 patients).^27^

Ferritin is the principal storage form of iron in the body. Thus, serum ferritin measurement can be used as a proxy of iron status in steady‐state individuals, and it is the most convenient laboratory test to estimate iron stores in resource‐limited settings. According to Olufemi et al.^23^ and Odunlade et al.,^24^ iron overload in Nigerian children with SCD is elevated beyond a ferritin level of 300 ng/ml. In Ghana, SCD remains a major public health concern where 2% (approximately 15,000) of all newborns have SCD and more than half of the patients are HbSS.^28^ While there has been more research into iron deficiency in patients with SCD, the burden of elevated iron stores or iron overload in sickle cell patients has not been investigated locally. In a holoendemic malaria setting like Ghana, children with SCD have an increased risk of multiple or chronic hemo transfusions which are mostly simple blood transfusions. Thus, these children may have an increased risk of elevated iron stores. To the best of our knowledge, this is the first time a study has investigated elevated iron stores among children with SCD in Ghana. The objectives of this study were to assess the extent of elevated iron stores and associated risk factors in a population of steady‐state children with SCD accessing care in a specialized SCD management clinic in Kumasi, Ghana. Data generated from this study would help clinicians to provide timely medical diagnoses and inform the management of elevated iron stores in children with SCD in the country.

## 2 METHODS

### 2.1 Study design

This was a hospital‐based cross‐sectional study that involved only quantitative methods of data collection and laboratory analysis of blood samples. The data was collected over a period of 4 months, from August to December 2021.

### 2.2 Study area

The study was conducted at the pediatric SCD clinic at the Komfo Anokye Teaching Hospital (KATH). KATH is a tertiary and major referral hospital located in the Kumasi Metropolis, the regional capital of the Ashanti region of Ghana.^29^ The hospital serves many areas in the middle, northern, and southern zones of the country, and it has a bed capacity of about 1200.^29^ The Pediatric SCD clinic is one of the largest SCD clinics in Ghana that provides outpatient services for children from 0 to 18 years.^29^ Until the recent decentralization of SCD clinics in the country, the pediatric SCD clinic in KATH was the only clinic serving the northern, middle, and southern zones, hence it has a well‐diversified population coverage.

### 2.3 Study population

The population targeted for the study were children with confirmed SCD attending a clinic visit at the pediatric SCD clinic at KATH. Children with SCD of all sexes, in a steady‐state and from the ages of 3 to 18 years were recruited and included in the study. Steady‐state was defined as the period when the patient with SCD is free of infection, pain, or other disease processes.^30^ However, all children with SCD who experienced VOPE in the last 3 months or currently experiencing inflammation, had been hemo transfused in the last 3 months before recruitment, and refused to give assent or the caregiver refused to consent were excluded from the study.

### 2.4 Sample size estimation and sampling technique

The minimum sample size estimated for the study was 177. This was determined using the Fisher's formula for sample size calculation, *N* = [z^2^p (1−p)]/d^2^,^31^ and based on the assumed prevalence of iron overload i.e. 21%, reported by Olufemi et al.^23^ in steady‐state children with SCD. Specifically, N was the minimum sample size estimated; z was the point of the standard normal distribution curve which was set at 1.96 (95% confidence interval [CI]); p was the assumed prevalence rate; and d was the degree of precision which was set at 6%. After review of medical records and complete blood count, 178 participants met the eligibility criteria and were included in the analysis.

Systematic random sampling technique was employed to recruit the study participants. SCD clinic registers were reviewed for the total number of patients who visited the clinic within the period of data collection. Using this number, the sampling frame (nth) was calculated from the quotient of the number of patients who visited the clinic (N) and the estimated sample size (n) i.e., nth = N/n.^32^ Therefore, participants were chosen at random after every “nth” count of patients visiting the clinic and in the subsequent days of the recruitment period.

### 2.5 Study procedure

The selected SCD patients were screened for eligibility. Permission was obtained from caregivers whose child met the inclusion criteria, and caregivers or patients more than 7 years were consented or assented respectively. Five milliliters of blood samples were collected from each patient, i.e., 2 and 3 ml were transferred into an ethylenediaminetetraacetic acid (EDTA) vacutainer and serum separator (SS) tube, respectively. The samples were placed in a cool box containing ice packs and transported to the Central Research Laboratory, KNUST, within 6–8 h of collection for processing. The fresh EDTA blood samples were subjected to complete blood count analysis using a hematology autoanalyzer (XN‐2000; Sysmex). Erythrocyte sedimentation rate (ESR) was also performed using the fresh EDTA blood samples to screen for possible inflammation. The fresh EDTA blood samples Following centrifugation of the sample in the SS tube, the serum was separated and stored in a minus 80°C freezer until a pool sample of 178 was obtained for the ferritin assay. Serum ferritin was measured by Ferritin enzyme immunoassay test kit (Fortress Diagnostics) according to the manufacturer's instructions.

### 2.6 Data collection

Caregivers and/or study participants were interviewed with an electronic semi‐structured questionnaire hosted on the School of Medicine and Dentistry, KNUST Research Electronic Data Capture (REDCap) server.^33^ REDCap is a secure web application for building and managing online surveys and databases. To ensure that quality data was collected, the questionnaire was answered by caregivers on behalf of study participants below the age of 13 years, while participants from 13 years and above responded to the questionnaire and in some cases were assisted by their caregivers. Moreover, questions were interpreted in the local language where necessary for easy comprehension. Medical records were reviewed for each study participant for the clinic visit to confirm their steady state and complete other relevant variables. The questionnaire was categorized into background characteristics, clinical characteristics, and current treatment. Furthermore, the laboratory results of study participants were entered into a Laboratory Documentation Sheet which was also hosted on REDCap so that each study participant has a point source document. The questionnaire was pilot tested at the pediatric SCD clinic in the Maternal and Child Health Hospital. A total of 10% of the calculated sample size was used for the pilot test to accomplish a reasonable power to ensure the reliability and validity of the questionnaire.

## 3 MEASUREMENT

### 3.1 Dependent/outcome variables

The outcome variable “elevated iron stores” was measured binary using serum ferritin ≤300 ng/ml and >300 ng/ml responses. Elevated iron stores were defined as serum ferritin >300 ng/ml according to previous studies^23^, ^24^, ^34^ Moreover, log transformation of serum ferritin concentration was carried out and used as the outcome variable in a linear regression analysis.

### 3.2 Independent/predictor variables

The predictor variables included demographic and clinical characteristics of study participants. Demographic characteristics such as age and gender were considered relevant in this study. The age of the study participants was categorized according to the WHO AGE categorization, i.e., <5 years, 5–9 years, 10–14 years, and ≥15 years.^35^ The clinical characteristics such as SCD genotype, VOPE in the last 12 months, frequency of hospitalization in the last 12 months, frequency of hemotransfusion in the last 12 months, ever been hemotransfused, history of chronic transfusion and hydroxyurea treatment were considered relevant for this study based on literature. Chronic transfusion was defined as receiving blood transfusions on a regular basis, i.e., at least one hemotransfusion in every month.^11^ Moreover, RBC, hemoglobin, hematocrit, mean cell volume, and mean cell hemoglobin were considered for the linear regression.

### 3.3 Statistical analysis

The data were analyzed using Stata (STATA/SE version 16.0). Descriptive statistics were performed for participant demographics, clinical, and laboratory characteristics. Categorical variables were expressed as frequency/percentage while continuous variables were expressed as mean/standard deviation and median/interquartile range (IQR) according to whether the distribution of the variables was Gaussian or not Gaussian, respectively. The Mann–Whitney test was computed to compare gender differences with regards to serum ferritin levels. The Kruskal–Wallis test was performed to compare age differences and SCD genotype with regards to serum ferritin levels. The relationship between serum ferritin levels and frequency of hemotransfusions per year was established by Kendall's correlation and graphically presented by a boxplot. The correlation between CBC indices and serum ferritin levels was established by Kendall's correlation. Moreover, a linear regression was used to predict the relationship between CBC indices and serum ferritin levels and graphically presented by a scatterplot. Bivariate and multivariate logistic regression analysis were performed to calculate the crude odds ratio and adjusted odds ratio at 95% CI between the dependent variable and all predictor variables. Statistical significance was considered at *p* < 0.05.

### 3.4 Ethical approval

Ethical clearance was obtained from the KATH Institutional Review Board (IRB) with reference: KATH IRB/AP/061/21. The background, aims, and study procedures were thoroughly explained to caregivers and patients where necessary in a language they can comprehend. Written informed consent and assent were obtained before any study participant was enrolled into the study.

## 4 RESULTS

### 4.1 Socio‐demographic and clinical characteristics of study participants

Table 1 describes the socio‐demographic characteristics of the study participants recruited for this study. In all, 178 participants were involved in this study. The age of the participants ranged from 3 to 18 years with a mean (*SD*) age of 9.6 (±4.3) years. Majority (34.3%) of the participants were within the ages of 5–9 years. Ninety (50.6%) of the participants were males. It was revealed that 45.5% of the participants were diagnosed by new‐born screening. Moreover, 69.7% of participants were diagnosed with the HbSS genotype. Eighteen (10.1%) of the respondents had experienced at least five VOPE and 8.4% had been hospitalized at least four times in the last 12 months. The major causes of hospitalization were VOPE (28.7%), other complications (28.7%), and anemia (22.9%). Majority (57.3%) of the participants have ever been hemotransfused, 16.3% have a history of chronic hemotransfusion, and 7.3% have been hemotransfused at least 3 times in the last 12 months. A hundred and eleven (62.4%) of the participants were on hydroxyurea treatment.

**Table 1:** Socio‐demographic and clinical characteristics of study participants

Variable	Frequency	Percentage (%)
Age (years)		
<5	28	15.73 (28/178)
5–9	61	34.27 (61/178)
10–14	59	33.15 (59/178)
≥15	30	16.85 (30/178)
**Mean (*SD*)**	**9.61 (±4.34)**	
Gender		
Female	88	49.44 (88/178)
Male	90	50.56 (90/178)
Diagnosis pathway		
New‐born screening	81	45.51 (81/178)
Nonnew‐born screening	97	54.49 (97/178)
SCD genotype		
HbSC	41	23.03 (41/178)
HbSS	124	69.66 (124/178)
Other	13	7.30 (13/178)
VOPE in the last 12 months		
0	76	42.70 (76/178)
1–4	84	47.19 (84/178)
≥5	18	10.11 (18/178)
Frequency of hospitalization in 12 months		
0	84	47.19 (84/178)
1	45	25.28 (45/178)
2	24	13.48 (24/178)
3	10	5.62 (10/178)
≥4	15	8.43 (15/178)
Cause of hospitalization in the last 12 months^a^		
VOPE	69	28.75 (69/240)
Anemia	55	22.92 (55/240)
Acute chest syndrome	22	9.17 (22/240)
Infection	18	7.50 (18/240)
Stroke	3	1.25 (3/240)
Congestive heart failure	2	0.83 (2/240)
Priapism	2	0.83 (2/240)
Other	69	28.75 (69/240)
Ever been hemotransfused		
No	76	42.70 (76/178)
Yes	102	57.30 (102/178)
History of chronic hemotransfusion		
No	149	83.71 (149/178)
Yes	29	16.29 (29/178)
Frequency of hemotransfusion in the last 12 months		
0	145	81.46 (145/178)
1–2	20	11.24 (20/178)
≥3	13	7.30 (13/178)
Hydroxyurea initiated		
No	67	37.64 (67/178)
Yes	111	62.36 (111/178)

*Note*: Other—Abdominal pain, Severe fever, Mesenteric crisis, Splenic sequestration, Jaundice, Liver impairment, Autoimmune hepatitis, Stuttering priapism.

Abbreviation: VOPE, vaso‐occlusive pain episode.

^a^ Multiple response.

### 4.2 Laboratory characteristics of study participants

Table 2 describes the complete blood count indices and serum ferritin threshold of the study participants. The median (IQR) RBC, hemoglobin (HGB), and hematocrit (HCT) were 2.70 10^6^/μl (2.40–3.47 10^6^/μl), 8.75 g/dl (7.80–9.70 g/dl) and 23.90% (21.60–26.80%), respectively. The mean (*SD*) mean cell haemoglobin (MCH), platelet (PLT), and white blood cell (WBC) was 30.66 pg (±4.73 pg), 358.46 10^3^/μl (±135.38 10^3^/μl) and 9.71 10^3^/μl (±3.043 10^3^/μl), respectively. The median serum ferritin was 129.55 ng/ml with an IQR of 68.95–240.26 ng/ml. 17.4% of participants had ferritin to be >300 ng/ml.

**Table 2:** Laboratory characteristics of study participants

Variable	Mean (±*SD*)/median (IQR)
RBC (10^6^/μl)^a^	2.70 (2.40–3.47)
HGB (g/dl)^a^	8.75 (7.80–9.70)
HCT (%)^a^	23.90 (21.60–26.80)
MCV (fL)^b^	84.46 (±11.55)
MCH (pg)^b^	30.66 (±4.73)
MCHC (g/dl)^a^	36.40 (35.20–37.30)
PLT (10^3^/μl)^b^	358.46 (±135.38)
WBC (10^3^/μl)^b^	9.71 (±3.043)
Lymph (10^3^/μl)^a^	4.07 (3.01–5.14)
Mono (10^3^/μl)^b^	0.92 (±0.41)
Neut (10^3^/μl)^a^	3.75 (2.73–5.11)
Eo (10^3^/μl)^a^	0.22 (0.11–0.46)
Baso (10^3^/μl)^a^	0.06 (0.04–0.09)

**Ferritin test**	***N*** = **178**
Ferritin (ng/ml)^a^	129.55 (68.95–240.26)
≤300	82.58 (147/178)
>300	17.42 (31/178)

Abbreviations: Baso, basophil; Eo, eosinophil; HCT, hematocrit; HGB, hemoglobin; IQR, interquartile range; Lymph, lymphocyte; MCH, mean cell hemoglobin; MCHC, mean cell hemoglobin concentration; MCV, mean cell volume; Mono, monocyte; Neut, neutrophil; PLT, platelet; RBC, red blood cell; *SD*, standard deviation; WBC, white blood cell.

a Median/IQR.

b Mean/*SD*.

### 4.3 Comparative serum ferritin levels for different gender, age, and SCD genotype of study participants

This section presents the findings on the differences of serum ferritin across gender and various age groups of the study participants. The median serum ferritin in the female participants was 146.49 ng/ml with an IQR of 86.53–273.16 ng/ml while the median serum ferritin in males was 117.58 ng/ml with an IQR of 65.26–216.32 ng/ml. The Mann–Whitney test showed that serum ferritin concentration for the two sexes were not significantly different (*p* = 0.074). The SCD children with SS, SC, and other genotype recorded median (IQR) serum ferritin of 111.14 (56.32–187.11), 135.43 (78.69–255.00), and 81.84 (50.00–178.00), respectively but the Kruskal–Wallis test revealed no significant difference between SCD genotype and serum ferritin concentration (*p* = 0.10). The median (IQR) serum ferritin among children under 5, 5–9 years, 10–14 years, and ≥15 was 131.97 ng/ml (77.43–230.52 ng/ml), 163.43 ng/ml (80.53–312.00 ng/ml), 94.00 ng/ml (59.74–165.71 ng/ml) and 166.86 ng/ml (88.86–243.68 ng/ml), respectively. The serum ferritin levels for the age groups were significantly different (*p* = 0.02) after performing a Kruskal–Wallis test. This was followed by the Conover‐Iman test and the pairwise comparison showed the main difference was between the age groups of 10–14 years and 5–9 years (*p* = 0.009) as summarized in Table 3.

**Table 3:** Pairwise comparison between serum ferritin levels and age of participants

Variable	*p* value			
**Age (years)**	**Diff (*p* value)**			
<5		<5	5–9	10–14
5–9	5–9	−1.30 (0.39)		
10–14	10–14	1.12 (0.40)	3.03 (0.009)	
≥15	≥15	−0.99 (0.32)	1.15 (0.44)	−2.31 (0.06)

### 4.4 Serum ferritin concentration varies with frequency of hemotransfusion in the last 12 months

**Figure 1:**
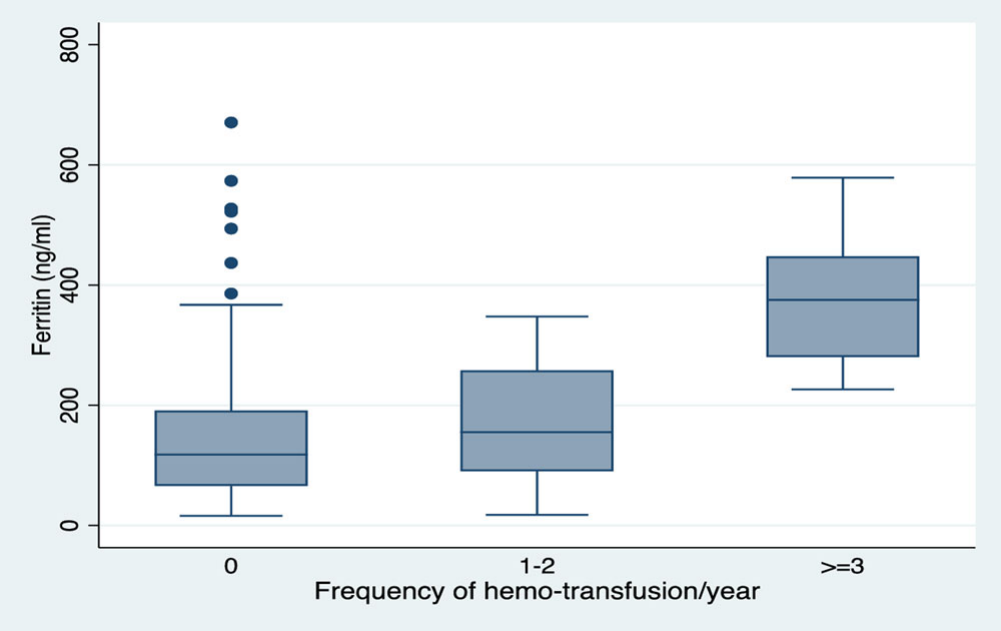
Variation of serum ferritin level as a function of the frequency of haemo transfusion during the last 12 months

The median (IQR) for participants who had received at least three hemotransfusions was 375.26 ng/ml (280.00–448.00 ng/ml), 155.23 ng/ml (90.26–258.03 ng/ml) for those who had received 1–2 hemotransfusion and 118.00 ng/ml (66.05–191.32 ng/ml) for those without any history of hemotransfusion in the last 12 months. Kendall's correlation was computed between serum ferritin concentration and frequency of hemotransfusion in the last 12 months and this revealed that serum ferritin levels increased proportionally with the frequency of hemotransfusions received in the last 12 months (*p* < 0.001). The distribution serum ferritin concentration and frequency of hemotransfusion in the last 12 months is presented in a boxplot as showed in Figure 1.

### 4.5 Red cell indices predict serum ferritin levels of steady‐state SCD children

**Figure 2:**
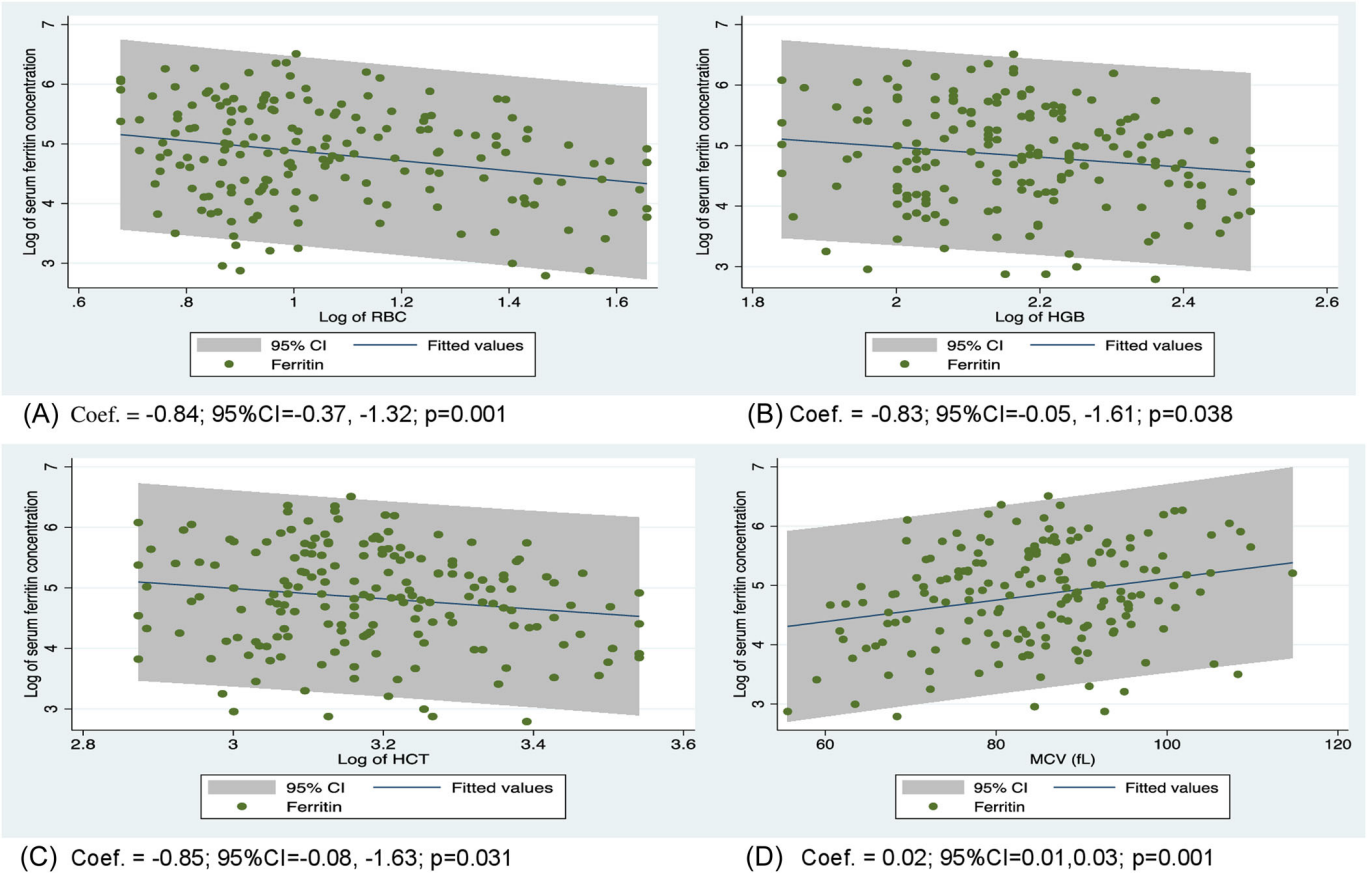
Linear relationship between serum ferritin levels and red blood indices of steady‐state SCD children. SCD, sickle cell disease.

The relationship between complete blood count and serum ferritin levels was investigated and this was computed by the Kendall's correlation test. It was shown that RBC (tau‐b = −0.14, *p* = 0.005), HGB (tau‐b = −0.11, *p* = 0.04) and HCT (tau‐b = −0.10, *p* = 0.04) were negatively correlated with serum ferritin levels. Further, the relationship between serum ferritin levels and mean cell volume (MCV) (tau‐b = 0.15, *p* = 0.004) and MCH (tau‐b = 0.14, *p* = 0.008) was positively correlated. On the contrary, there was no significant correlation between serum ferritin levels and the differential white blood cell counts (Supporting Information). Improving the diagnosis of elevated iron stores is critical for effective monitoring. Therefore, it was important to further establish the association between the RBC indices and serum ferritin levels so it can aid physicians to predict elevated iron stores in SCD children. This was achieved through linear regression analysis and it was revealed that RBC, HGB, HCT, MCV, and MCH could reliably predict serum ferritin levels. For every unit increase in RBC, HGB, and HCT, serum ferritin concentration decreases by 0.84 ng/ml (Coef. = −0.84; 95% CI = −0.37, −1.32; *p* = 0.001), 0.83 ng/ml (Coef. = −0.83; 95% CI = −0.05, −1.61; *p* = 0.04) and 0.85 ng/ml (Coef. = −0.85; 95% CI = −0.08, −1.63; *p* = 0.03), respectively. However, the serum ferritin concentration increased by 0.02 ng/ml (Coef. = 0.02; 95% CI = 0.01, 0.03; *p* = 0.001) and 0.04 ng/ml (Coef. = 0.04; 95% CI = 0.01, 0.07; *p* = 0.002) for every unit increase of MCV and MCH, respectively. This is presented in Figure 2.

### 4.6 Factors associated with elevated iron stores among study participants

To establish the risk factors associated with elevated iron stores among the study participants, a crude analysis was performed. Here, the data revealed that the frequency of hospitalization in the last 12 months, ever been hemo‐transfused, chronic hemotransfusion and frequency of hemotransfusion in the last 12 months were significant factors associated with elevated iron stores in steady‐state SCD children. The multivariate logistic regression revealed that male SCD patients had reduced‐odds of having elevated iron stores (adjusted odds ratio [aOR] = 0.35; 95% Cl = 0.18–0.96; *p* = 0.03) compared to female SCD patients. SCD children who had ever been hemo‐transfused had increased‐odds of having elevated iron stores (aOR = 8.72; 95% CI = 1.89–30.19; *p* = 0.006) compared with their counterparts. History of chronic hemotransfusion increased the odds of having elevated iron stores (aOR = 11.41; 95% Cl = 3.11–30.85; *p* < 0.001) compared to SCD patients without a history of chronic hemotransfusion. However, SCD patients on hydroxyurea treatment had reduced‐odds of having elevated iron stores (aOR = 0.18; 95% CI = 0.06–0.602; *p* = 0.006) compared to those who had not initiated hydroxyurea treatment as described in Table 4.

**Table 4:** Risk factors associated with high ferritin among the participants

Variable	Ferritin ng/ml		OR (95% Cl)	*p* value	aOR (95% CI)	*p* value
	<300% (*n*/*N*)	≥300% (*n*/*N*)				
Age						
<5	89.29 (25/28)	10.71 (3/28)	Ref		Ref	
5–9	72.13 (44/61)	27.87 (17/61)	3.22 (0.86–12.08)	0.08	2.35 (0.45–12.30)	0.31
10–14	89.83 (53/59)	10.17 (6/59)	0.94 (0.22–4.08)	0.94	0.35 (0.06–2.18)	0.26
≥15	83.33 (25/30)	16.67 (5/30)	1.67 (0.36–7.74)	0.51	0.27 (0.03–2.21)	0.22
Gender						
Female	72.70 (69/88)	15.30 (19/88)	Ref		Ref	
Male	74.30 (78/90)	15.70 (12/90)	0.56 (0.25–1.23)	0.15	0.33 (0.12–0.90)	0.03
SCD genotype						
Other	84.62 (11/13)	15.38 (2/13)	Ref		Ref	
HbSS	81.45 (101/124)	18.55 (23/124)	1.25 (0.26–6.04	0.78	1.17 (0.12–11.14)	0.89
HbSC	85.37 (35/41)	14.63 (6/41)	0.94 (0.17–5.36	0.95	1.27 (0.13–12.71)	0.84
VOPE in the last 12 months						
0	82.89 (63/76)	17.11 (13/76)	Ref		Ref	
1–2	89.83 (53/59)	10.17 (6/59)	0.55 (0.19–1.54)	0.26	0.29 (0.07–1.19)	0.09
3–4	72.00 (18/25)	28.00 (7/25)	1.88 (0.65–5.43)	0.24	0.99 (0.19–4.87)	0.99
≥5	72.22 (13/18)	27.78 (5/18)	1.86 (0.57–6.14)	0.31	1.16 (0.17–7.76)	0.88
Frequency of hospitalization in 12 months						
0	88.10 (74/84)	11.90 (10/84)	Ref		Ref	
1	84.44 (38/45)	15.56 (7/45)	1.36 (0.480–3.87)	0.56	0.54 (0.12–2.30)	0.40
2	75.00 (18/24)	25.00 (6/24)	2.47 (0.79–7.68)	0.12	0.62 (0.09–4.50)	0.63
3	100.00 (10/10)	0.00 (0/0)	1.00	–	1.00	–
≥4	46.67 (7/15)	53.33 (8/15)	8.46 (2.52–28.37)	0.001	0.45 (0.03–6.68)	0.57
Ever been hemotransfused						
No	94.74 (72/76)	5.26 (4/76)	Ref		Ref	
Yes	73.53 (75/102)	26.47 (27/102)	6.48 (2.16–19.44)	0.001	8.72 (1.89–30.19)	0.006
History chronic hemotransfusion						
No	89.93 (134/149)	10.07 (15/149)	ref		Ref	
Yes	44.83 (13/29)	55.17 (16/29)	10.99 (4.45–27.20)	<0.001	11.41 (3.11–30.85)	<0.001
Frequency of hemotransfusion in the last 12 months						
0	86.99 (107/123)	13.01 (16/123)	Ref		Ref	
1–2	90.91 (30/33)	9.09 (3/33)	2.35 (0.76–7.25)	0.14	0.31 (0.50–1.86)	0.20
≥3	45.46 (10/22)	54.54 (12/22)	8.03 (2.98–21.60)	<0.001	1.79 (0.27–11.95)	0.55
Hydroxyurea initiated						
No	79.11 (53/67)	20.89 (14/67)	Ref		Ref	
Yes	84.69 (94/111)	15.31 (17/111)	0.69 (0.31–1.49)	0.34	0.18 (0.06–0.62)	0.006

Abbreviations: aOR, adjusted odds ratio; CI, confidence interval; OR: odds ratio.

## 5 DISCUSSION

The diagnosis of iron overload in children with SCD is not a common practice in Ghana. In the present study, we evaluated the levels of iron stores among children with SCD using serum ferritin as the proxy of measurement. Serum ferritin is gender and age dependent, and thus, it may vary according to the gender and age group of individuals. Comparing iron stores between male and female SCD children, the median serum ferritin was higher among females than males, but this difference was not significant. Similarly, high mean serum ferritin was reported among females than males by Akodu et al.,^23^ however, the mean difference was statistically significant unlike our findings. The difference may be attributed to variations in the age of the study population and the distribution which were under five SCD children and Gaussian distribution, respectively, in the case of Akodu et al.^23^ However, our findings were consistent with Makulo et al.^25^ since their study had similar study design, age of the study population, and Gaussian distribution. Furthermore, there was a median difference in serum ferritin levels among the age categories of the participants. The pairwise comparison showed that the median difference was dominantly observed between participants from the ages of 10–14 years and 5–9 years. Unlike our findings, Makulo et al.^25^ found no significant difference in serum ferritin levels between the various age categories of children with SCD and this may be due to the smaller sample size of their study (*N* = 70). Our findings confirm that serum ferritin level in children with SCD is a function of age.

Hematological parameters are crucial in disease diagnosis or prognosis because changes in these parameters above or below normal range may account for clinical complications in patients. In SCD patients, hematological parameters are evaluated regularly on clinic visits to ensure good management of the disease. Thus, we believe it is imperative to examine the association between serum ferritin and hematological parameters so that it can inform physicians when making decisions on iron store diagnosis. The study found no correlation between serum ferritin and differential white blood cell counts. These findings were consistent with the reports of several studies that were conducted on children with SCD and other chronic non‐communicable diseases^25^, ^36^, ^37^ Ferritin is an acute‐phase protein that is elevated in the presence of infection or inflammation. Differential white blood cells are major components of the body's defensive system that may become abnormal in the presence of an infection or disease. Thus, there is a positive correlation between serum ferritin and the differentials of white blood cells. However, we anticipated the observations of the present study since the participants recruited for the current study were steady‐state SCD children. Thus, Onabanjo et al.^36^ concluded that there are no associations between iron status and the differentials of white blood counts in steady‐state children.

Furthermore, the study revealed that decreasing RBC, HGB, and HCT levels were significantly correlated with increasing levels of serum ferritin and vice versa. Iron is the chief component of HGB which is found in RBCs and this helps to circulate oxygen throughout the body of an individual. The relationship observed between RBC, HGB, and serum ferritin levels in this study may be due to the hemolysis of RBC and an excessive breakdown of HGB, which commonly occurs in patients with SCD. Consequently, an excessive amount of iron is released into the blood which may cause a rise in serum ferritin concentration. Studies have shown that low or decreasing HCT (HCT reflects the percentage of blood volume composed of RBCs) implies that enough RBCs are not being produced or RBCs are hemolyzing, thereby more iron is released into the blood^38^, ^39^ This may explain the correlation observed between serum ferritin levels and HCT in the current study. Unlike our findings, several studies did not find significant correlations between serum ferritin and HCT^25^, ^40^ RBC and HGB^24^, ^37^, ^41^ levels in children with SCD. The differences may be attributed to variations in the sample size, age of the study population, and study designs.

Nonetheless, the study also revealed that serum ferritin concentration significantly increases when MCV and MCH levels increase and vice versa. The positive trend observed in our study was consistent with the findings of Gomez et al.,^37^ however, the correlations reported by these authors were not significant. The discrepancy between these two findings could be attributed to the study design and sample size difference, which was a prospective cohort with iron supplement intervention and 141participant (73 SCD patients and 68 non‐SCD patients), respectively in the case of Gomez et al.^37^ Nonetheless, Fiorelli^42^ in an editorial explained that the positive correlation between serum ferritin levels and MCV and MCH levels may reflect increased iron uptake and hemoglobin synthesis by immature erythroid cells. Thus, it was expected that MCH and MCV would correlate positively with serum ferritin in the current study.

In malaria‐endemic settings such as Ghana, SCD patients have an increased risk of chronic hemolysis, iron recycling, and anemia leading to recurrent hemotransfusions. As a result, SCD patients may experience elevation of iron stores. In the present study, the majority of SCD children have ever been hemotransfused, which concur with the findings of several studies^24^, ^25^, ^43^ Furthermore, we found that the increasing number of hemotransfusions significantly increased serum ferritin concentration. Specifically, it was demonstrated that SCD children who had received at least three hemotransfusions have higher median serum ferritin than those who received 1–2 and no hemotransfusions in that order, and the median difference was statistically significant. Our findings corroborate with the reports of several studies^25^, ^44^, ^45^ however, it contrasts with the outcome of Gomez et al.^37^ and Odunlade et al.^24^ who did not find a significant correlation between the number of blood transfusions received and serum ferritin concentration. We expected the outcome in the present study because during hemotransfusion, about 200 mg of iron is released into the body and only about 1 mg iron is eliminated through the intestinal epithelial and the skin every day^14^, ^22^ Since the body lacks a robust physiologic system for iron elimination, iron builds up in the body and thereby, increases the levels of serum ferritin in hemotransfused SCD patients. Although SCD children who had received at least three hemotransfusions have a ferritin level greater than the median serum ferritin, some of the SCD children who had received 1–2 hemotransfusion or who had not received hemotransfusion in the last 12 months recorded high serum ferritin concentration. This observation may suggest that besides hemotransfusion, the elevation of iron stores in some of our study participants may be due to recurrent intravascular hemolysis in the past, which is reported to predispose SCD patients to excessive gastrointestinal absorption of iron.^14^

Several studies have reported the prevalence of elevated iron stores in SCD children in Africa and it ranges from 22% to 71%^23^, ^24^, ^25^, ^46^ In contrast, the prevalence of elevated iron stores was 17% among SCD children in Ghana according to the present study which is below the preponderances previously reported. The low prevalence relative to the other studies may be attributed to the differences in the age of the study population, sample size, genetic factors, and definition of elevated iron stores using ferritin as the proxy of measurement. Most importantly, serum ferritin level beyond 300 ng/ml is predictive of elevated iron stores^23^, ^24^ however, the definition of elevated iron stores in some of these studies was beyond 500 ng/ml or 1000 ng/ml^25^, ^46^ Furthermore, three or more hemotransfusions and being hospitalized for at least four times in the last 12 months were likely to cause elevated iron stores in SCD children in the present study. These findings were similar to the reports of other studies^25^, ^47^ We anticipated these observations because the more an SCD patient is hospitalized, the likelihood the patient will be hemotransfused, given that SCD patients have an increased risk of hemotransfusion.

Moreover, the present study showed that ever been hemotransfused and a history of chronic hemotransfusion was significantly associated with elevated iron stores and this is consistent with several studies^45^, ^47^, ^48^ When adjusted for all covariates, children with SCD who had ever been hemotransfused and chronically transfused in the past were about 9 and 11 times likely to have elevated iron stores, respectively. Eveline et al. showed that iron overload is more concerned with the male gender and this is so because blood loss during menstruation results in significant iron loss in females, unlike their male counterparts who have no robust mechanism to remove iron from the body.^49^ However, this was not the scenario in our study as we observed males have reduced odds of developing elevated iron stores. This is interesting and warrants further investigation to unravel the underlying mechanisms. On the other hand, SCD children who were on hydroxyurea treatment had reduced odds of developing elevated iron stores. These findings were similar to the report by Italia et al.^50^ which showed that serum ferritin decreased significantly in SCD patients after 2 years of hydroxyurea therapy and also in mice models treated with hydroxyurea.^51^ Makulo et al.^25^ found no significant association between elevated iron stores and hydroxyurea treatment in SCD children. The differences relative to the results of other studies could be attributed to compliance with hydroxyurea treatment. It is reported that hydroxyurea is a radical scavenger that has hydroxamate function properties and hence can act as an iron chelator. This may explain the observation in the present study; however, we strongly suggest further studies to provide more insight into the iron chelation properties of hydroxyurea.

## 6 LIMITATIONS

This study provided valuable information that may be used by local physicians to monitor elevated iron stores or iron overload in children with SCD. However, the study has some limitations that can strengthen our findings when they are addressed in future studies. First, we did not perform liver biopsy or magnetic resonance interference which has been shown to provide an excellent assessment of elevated iron stores (iron overload)^43^, ^45^ to confirm our findings. Second, serum creatinine reactive protein levels were not measured to confirm the steady state of the study participants due to limited funds. However, this was complemented by reviewing the medical records of the participants clinic visit and performing ESR test to further screen those who were recruited. Third, the findings could have been strengthened by providing information on the products of blood transfused (whole blood, pack‐cell, and plasma) and the exact volume of blood received by patients. However, some of these factors were not available in the medical records of some study participants and some were also not hemotransfused at the facility, hence these factors were not available for them.

## 7 CONCLUSION

The magnitude of elevated iron stores is high among children with SCD in the present study and this must be given the needed attention. SCD children who have a history of chronic hemotransfusion or had received at least three hemotransfusions in a year should be monitored for elevated iron stores. Red cell indices can provide invaluable information regarding the risk of iron store elevation and can serve as a prompt for physicians to monitor the elevation of iron stores in SCD children. We suggest further studies that take into consideration the limitations of this present study and a detailed study on the iron chelation effect of hydroxyurea on iron stores in SCD patients.

## AUTHOR CONTRIBUTIONS

**Ernest Amanor**: Conceptualization; Data curation; Formal analysis; Investigation; Methodology; Writing – original draft; Writing – review & editing. **Alexander Kwarteng**: Investigation; Supervision; Writing – review & editing. **Amma Larbi**: Supervision; Writing – review & editing. **Fatima Amponsah Fordjour**: Data curation; Methodology; Writing – review & editing. **Kelvin Kwaku Koranteng**: Data curation; Methodology. **David Sebbie Sackey**: Methodology; Resources; Writing – review & editing. **Emmanuel Bannor**: Methodology; Resources; Writing – original draft. **Francis Adjei Osei**: Conceptualization; Methodology; Writing – review & editing. **Aliyu Mohammed**: Formal analysis; Methodology; Validation; Writing – review & editing. **Ezekiel Bonwin Ackah**: Methodology; Resources; Validation; Writing – review & editing. **Samuel Frimpong Odoom**: Data curation; Formal analysis; Methodology; Validation; Writing – review & editing. **Samuel Blay Nguah**: Formal analysis; Methodology; Validation; Writing – review & editing. **Vivian Paintsil**: Funding acquisition; Project administration; Supervision; Writing – review & editing. **Alex Osei‐Akoto**: Funding acquisition; Project administration; Supervision; Writing – review & editing.

## CONFLICTS OF INTEREST

The authors declare no conflicts of interest.

## TRANSPARENCY STATEMENT

The lead author Ernest Amanor affirms that this manuscript is an honest, accurate, and transparent account of the study being reported; that no important aspects of the study have been omitted; and that any discrepancies from the study as planned (and, if relevant, registered) have been explained.

## Supporting information

Supplementary information.

Supplementary information.

## Data Availability

The data and materials are available in the corresponding author's institution and will be made available upon formal request.
