# Supplementary material for: Iron stores in steady‐state sickle cell disease children accessing care at a sickle cell disease clinic in Kumasi, Ghana: A cross‐sectional study
Source: Health Sci Rep. 2022 Nov 24;5(6):e934. doi: 10.1002/hsr2.934 (PMC9686355; doi:10.1002/hsr2.934)
Supplement: Supplementary file 2 — Supplementary information. [file HSR2-5-e934-s001.docx]

**Supplementary material**

This consist of data describing the relationship between the complete blood count indices including the differential white blood cell counts and the serum ferritin concentration.
